# Risk-taking influences perceived dominance, prestige, and leadership endorsement in Japanese adults

**DOI:** 10.3389/fpsyg.2025.1529892

**Published:** 2025-04-23

**Authors:** Akira Ono, Xianwei Meng

**Affiliations:** Graduate School of Informatics, Nagoya University, Nagoya, Aichi, Japan

**Keywords:** leadership endorsement, regulatory focus theory, risk-taking, dominance, prestige, costly signaling theory

## Abstract

Risk-taking behavior occurs everywhere in our social lives, but little is known about how it is socially evaluated. Previous research has shown that risk-taking functions as a signal of a risk-taker’s dominance and prestige, increasing their likelihood of being endorsed as a leader in intergroup competitive contexts. However, the findings were obtained from Western cultures, leaving it unclear how these social evaluations are made in other cultures. This study investigated the social evaluations of risk-takers among Japanese individuals, who are rooted in Eastern culture which has been known that many social norms and traditions differ from Western cultures. Through a survey-based investigation (*N* = 299), we found that while risk-takers are perceived as more dominant, there was no difference in prestige evaluation between risk-takers and risk-avoiders. Moreover, leadership endorsement varies across contexts, with risk-taking increasing endorsement in competitive situations but decreasing it in cooperative ones, mediated by perceived dominance. These findings not only clarified the social evaluation of risk-taking behavior in one of the Eastern cultures but also provided insights into nuanced perceptions of risk-takers across diverse cultural settings.

## Introduction

In Western culture, risk-takers are often perceived as more dominant and prestigious than risk-avoiders, and more capable in terms of leadership in intergroup competitive situations (van Kleef et al., 2021). Building on van Kleef et al.’s study, the present research aims to examine whether these evaluations can also be observed in a Japanese sample, which is rooted in Eastern cultural traditions. This will help determine whether culture (e.g., social norms) serves as a significant factor in shaping the perception of risk-takers.

### Risk-taking shapes perceived dominance, prestige, and leadership in Western culture

How risk-taking influences the social evaluations of risk-takers may be predicted based on Costly Signaling Theory (CST). CST posits that actions that impose potential costs on the actor serve as signals of underlying qualities that are difficult or impossible to directly observe (Zahavi, 1995). CST has been widely used to explain how costly behaviors could lead to social influence in human interactions. For instance, according to CST, donations to public goods serve as a signal of the contributor’s dedication to the group (Gintis et al., 2001), and the benefit brought about by the signal is an enhancement of reputation within the group, which may result in an improvement of status within the group (Van Vugt and Hardy, 2010). Moreover, punishment serves as a signal to indicate the punisher’s formidability because punishing others comes with the cost of retribution, and only those who can endure it are likely to engage in such behavior (Gordon et al., 2014). As risk-taking inherently involves potential costs, CST may predict that such behaviors serve as signals of the underlying qualities (e.g., formidability) of risk-takers.

Following this logic, van Kleef et al. (2021) explored the social evaluations of risk-takers concerning dominance, prestige, and leadership with participants in the Netherlands. Dominance and prestige represent two fundamental pathways to attaining social rank within groups (Cheng et al., 2013). Dominance involves the use of violence, coercion, threats, and punishment to exert control over subordinates and achieve high status and influence. In contrast, prestige entails the demonstration of skills, abilities, and knowledge valued by the group to gain respect and attain high status and influence.

In van Kleef et al. (2021; Exp. 3), participants were presented with profiles describing individuals as either risk-takers or risk-avoiders. Participants were then asked to evaluate the levels of dominance and prestige attributed to these individuals and to indicate the extent to which they would endorse them as leaders in both intergroup competitive and cooperative contexts. The results revealed that participants perceived risk-takers as possessing higher levels of dominance and prestige than risk-avoiders. Moreover, risk-takers were more likely to be endorsed as leaders in intergroup competitive scenarios than risk-avoiders. These findings suggest that risk-taking serves as a signal conveying information about an individual’s potential aptitude for attaining a higher social rank within a group.

### Limited investigations in Eastern cultures

van Kleef et al. (2021) have provided significant insights into the perception of risk-takers. However, this investigation was conducted within a Western cultural context (i.e., the Netherlands) and it remains an open question whether individuals in Eastern cultures evaluate risk-takers in a manner similar to or different from those in the Western culture.

Studies have repeatedly shown that social recognition varies across cultures, with a typical example being the contrast between Western and Eastern societies. For instance, Markus and Kitayama (1991) classify self-construal into “independent self-construal” and “interdependent self-construal.” The former, prevalent in Western cultures, defines individuals based on unique attributes that distinguish them from others, whereas the latter, common in Eastern cultures, conceptualizes individuals through their relationships and group memberships. Furthermore, Torelli et al. (2014) utilize a framework of individualism (common in Western cultures) and collectivism (common in Eastern cultures) to explain cultural differences in values. In Western cultures, personal goals, achievement, and autonomy are emphasized (Hofstede, 2001; Triandis, 1995), and individuals tend to associate competence (e.g., efficacy, confidence) with high status (Torelli et al., 2014). In contrast, in Eastern cultures, group goals, loyalty, and sociability are valued (Hofstede, 2001; Triandis, 1995), and individuals associate warmth (e.g., generosity, kindness) with high status (Torelli et al., 2014).

These cultural differences may reflect broader philosophical traditions and social norms, which are likely to differ between Western and Eastern cultures (Confucius, 1980; Cooper and Hutchinson, 1997; Kim and Markus, 1999; Kinias et al., 2014; Locke, 1847). Particularly relevant to the current topic, the traits associated with high status may differ between Western and Eastern cultures. In Western culture, where individual uniqueness is more likely to be valued (Hofstede, 2001; Markus and Kitayama, 1991; Triandis, 1995), two traits that reliably lead to greater status are extraversion (Anderson et al., 2001) and dominance (Cheng et al., 2013). In contrast, in Eastern cultures, status is often accorded to those who prioritize group harmony and integration (Markus and Kitayama, 1991; Torelli et al., 2014). Thus, traits valued for status in Western cultures may be viewed unfavorably and, in some cases, even cause status loss in Eastern cultures (Kim and Pettit, 2019).

Addtionally, much of the research on social cognition (e.g., fairness and cooperation) has been conducted in WEIRD – Western, Educated, Industrial, Rich, Democracies–societies (Henrich et al., 2010). It is noteworthy that only 12% of the world’s population resides in such regions, and the findings established thus far may not be replicable outside of these industrialized Western contexts (Henrich et al., 2010).

Given the cultural differences between Western and Eastern societies, as well as the possibility that findings from WEIRD populations may not be replicable in Eastern cultures, it is crucial to investigate whether the social evaluation of risk-takers observed in Western contexts applies similarly to Eastern cultures and to explore the cultural factors that shape these perceptions.

### Social evaluation of risk-takers across Western and Eastern cultures: dominance and prestige

We propose that cultural differences or similarities between Western and Eastern societies in the social evaluation of risk-takers may depend on domains, such as prestige and dominance.

#### Regulatory focus and risk-taking

The possible cultural differences may derive from Regulatory Focus Theory (RFT). RFT distinguishes between promotion focus and prevention focus (Higgins, 1997). Individuals oriented toward promotion focus pursue personal aspirations and ideals, have a high sensitivity to positive outcomes, and employ eager strategies to achieve goals. Whereas those oriented toward prevention focus pay attention to safety, duty, and responsibility, have a high sensitivity to negative outcomes, and tend to use vigilant strategies to achieve goals (Higgins, 1997, 2000). Moreover, individuals who employ the eager strategy stemming from a promotion focus are willing to take risks to maximize their benefits, whereas those who use the vigilant strategy stemming from a prevention focus are reluctant to take risks to minimize their losses (Crowe and Higgins, 1997).

Previous studies have indicated that regulatory focus varies between Western and Eastern cultures (Heine et al., 2001; Lockwood et al., 2005), possibly stemming from self-construal patterns (Lee et al., 2000). Westerners, who predominantly exhibit an independent self-construal, emphasize the positive aspects of the self and potential gains to distinguish themselves favorably from others. In contrast, Easterners, who primarily exhibit an interdependent self-construal, prioritize fulfilling obligations and avoiding risks to maintain social connections. Consequently, Westerners are more likely to adopt a promotion focus, whereas Easterners tend to adopt a prevention focus (Lee et al., 2000). Indeed, Lee et al. (2000) found that Westerners perceive promotion-focused information as more important than prevention-focused information, whereas Easterners exhibit the opposite pattern. Furthermore, Westerners are generally motivated by positive role models, whereas Easterners are more likely to be influenced by negative role models (Lockwood et al., 2005). These findings suggest that attitudes toward risk-taking may differ between Western and Eastern cultures, which may in turn influence the social evaluation of risk-takers across cultural contexts.

#### Hypothesis on the prestige evaluation of risk-takers

As valuable knowledge, skills, and experiences vary across fields, groups, and cultures, the evaluation of prestige is likely to be context-dependent (Henrich and Gil-White, 2001). For instance, an individual highly respected in the field of science may not necessarily hold the same level of esteem in the field of sports. Given this variability, the evaluation of risk-takers’ prestige may differ across cultures, depending on how each culture perceives the social value of risk-taking.

Based on cultural differences in regulatory focus, Westerners, who are oriented toward a promotion focus, emphasize growth, achievement, and potential gains, which may lead them to perceive risk-taking as a valuable means of attaining success (Lee et al., 2000). Consequently, risk-takers may be regarded as more prestigious than risk-avoiders. In contrast, Easterners, who are oriented toward a prevention focus, prioritize risk avoidance and failure prevention to maintain group harmony, as risk-taking may be perceived as a potential threat to social stability in Eastern cultures (Heine et al., 1999). As a result, risk-takers may not be considered more prestigious than risk-avoiders in Eastern cultures.

*H1*: There is no significant difference in prestige evaluations of risk-takers and risk-avoiders in Eastern cultures.

#### Hypothesis on the dominance evaluation of risk-takers

However, we predict that people in Eastern cultures will perceive risk-takers as more dominant than risk-avoiders, similar to findings in Western cultures (van Kleef et al., 2021). The evaluation of dominance is likely independent of specific domains and cultural backgrounds (Henrich and Gil-White, 2001). For instance, body size is often considered a cross-culturally relevant cue for dominance. In non-human animals, individuals with larger body sizes tend to derive greater benefits from strength and power (Brown and Maurer, 1986). A similar pattern has even been observed in human infants, where a larger body size is associated with perceptions of dominance (Thomsen et al., 2011). Previous research has demonstrated that risk-takers are more likely to be perceived as having a larger body size, greater muscularity, and a higher propensity for aggression than risk-avoiders (Fessler et al., 2014). Notably, this phenomenon has been replicated in two distinct cultural contexts, the United States and Fiji, suggesting a degree of cross-cultural consistency. These findings suggest that risk-takers are more likely to be perceived as dominant, and this perception appears to hold across different cultural settings.

*H2*: Risk-takers are more likely to be perceived as dominant than risk-avoiders in Eastern cultures.

### Leadership endorsement of risk-takers across Western and Eastern cultures: intergroup competitive and cooperative situations

#### A mediation analysis to identify mechanisms of leadership endorsement of risk-takers

To clarify the mechanisms underlying the relationship between risk-taking and leadership endorsement in Eastern cultures, a mediation analysis using dominance and prestige as mediating variables would be particularly insightful. In van Kleef et al. (2021; Exp. 3), the perceived prestige of risk-takers positively mediated leadership endorsement in both intergroup competitive and cooperative contexts. Conversely, the perceived dominance of risk-takers positively mediated leadership endorsement in intergroup competitive contexts but negatively mediated leadership endorsement in intergroup cooperative contexts (although this effect was not statistically significant).

Given that prestige evaluations may not be influenced by risk-taking demonstrations in Eastern cultures we did not expect a mediating effect of prestige. However, the perceived dominance of risk-takers is expected to play a significant role in influencing leadership endorsement across both contexts. More specific hypotheses regarding the mediating effect of perceived dominance are outlined below.

#### The possible mediating effect of risk-takers’ perceived dominance on their leadership endorsement in intergroup competitive situations

In intergroup competitive situations, individuals are more likely to endorse dominant leaders, as these leaders are perceived to maintain or create advantages over competing groups for the benefit of the group as a whole (Hasty and Maner, 2025; Little et al., 2007; Spisak et al., 2012a; Spisak et al., 2012b). This assertion is supported by several empirical studies. For instance, individuals with dominant traits (e.g., masculine facial features) are more likely to be endorsed as leaders in intergroup competitive contexts (Spisak et al., 2012b). Moreover, leadership endorsements of dominant individuals are mediated by perceptions of their ability to enforce collective action (e.g., by punishing free-riders) and to defeat rival groups (Hasty and Maner, 2025). As we expect risk-takers to be perceived as more dominant than risk-avoiders in Eastern cultures, we hypothesized that they would be more likely to be endorsed as leaders in intergroup competitive situations.

*H3*: In Eastern cultures, risk-takers are more likely than risk-avoiders to be endorsed as leaders in competitive situations, positively mediated by dominance.

#### The possible mediating effect of risk-takers’ perceived dominance on their leadership endorsement in intergroup cooperative situations

In intergroup cooperative contexts, individuals who are capable of maintaining and fostering positive intergroup relationships based on empathy, altruism, and reciprocity are more likely to be supported as leaders in promoting beneficial cooperation between groups. For example, individuals exhibiting feminine characteristics are often preferred as leaders over those displaying dominant traits in cooperative contexts (Spisak et al., 2012b). Moreover, dominant individuals are often prone to abusing their power and prioritizing their own preferences over group goals (Maner and Mead, 2010). In such cases, being perceived as dominant may negatively impact leadership endorsement in cooperative contexts. In Eastern cultures, risk-takers may be perceived as dominant, which can, in turn, decrease leadership endorsement in intergroup cooperative situations.

*H4*: In Eastern cultures, risk-takers are less likely than risk-avoiders to be endorsed as leaders in cooperative situations, negatively mediated by dominance.

## Method

### Participants

We recruited 300 Japanese adults. The required sample size was determined prior to data collection using G*Power (Faul et al., 2007), based on the effect of condition (i.e., high or low risk) on prestige evaluation, as reported in van Kleef et al. (2021; Exp. 3). A power analysis indicated that a minimum of 266 participants was necessary to detect a potential effect using an independent-samples t-test, assuming the effect size for prestige reported in van Kleef et al. (2021; Exp. 3) (*d* = 0.40), a significance level of *α* = 0.05, and a statistical power of 1 – *β* = 0.90. The effect size for prestige was selected because it was smaller than that observed for dominance in the van Kleef et al. (2021; Exp. 3) study^1^. However, to account for potential exclusions (e.g., mismatches between participant IDs and web-recorded IDs), we aimed to recruit 300 participants. Participants were recruited through CrowdWorks, a crowdsourcing management company. One participant was excluded because the ID recorded on the questionnaire did not match the ID assigned by CrowdWorks. The final sample comprised 299 Japanese adults (154 men, 143 women, 2 others; M_age_ = 39.78, SD = 10.07, Age range = 19–73).

We confirmed that the current sample accurately represents the regional diversity of the Japanese population, meaning that it aligns with the population distribution across Japan and is not biased toward specific regions. To verify this, we calculated the proportion of participants by prefecture and compared it with Japan’s national population distribution (Ministry of Internal Affairs and Communications, 2024). The analysis revealed a strong positive correlation (Pearson’s *r* = 0.94, *p* < 0.001), indicating that the regional distribution of our participants closely mirrors that of the national population.

Prior to accessing the survey, participants read an informed consent statement outlining that participation was voluntary and that they could withdraw at any time. Participants were considered to have consented to the study upon clicking the “agree” button. Our survey protocol received approval from the Ethics Committee of the Department of Cognitive and Psychological Sciences at Nagoya University (No. 240105-C-03-1).

### Experimental design

This study used a 2 × 2 mixed design, incorporating both between-subjects and within-subjects factors. The between-subjects factor, risk-taking, consisted of two conditions: high-risk and low-risk. Participants were randomly assigned to one of these conditions. The within-subjects factor, the group relationship context of leadership endorsement, included two conditions: competitive and cooperative intergroup situations.

### Manipulations and measures

#### Manipulating risk-taking

The manipulation of risk-taking was nearly identical to that of van Kleef et al. (2021; Exp. 3). Participants were presented with a scenario describing the profile of a target individual named Hikaru (see Supplementary file). Hikaru was described as either a risk-taker or a risk-avoider, depending on the condition to which participants were assigned. We chose the name “Hikaru” as it is gender-neutral in Japan, intending to exclude possible gender effects of risk-takers and risk-avoiders. In the high-risk condition, Hikaru’s life motto was “If you do not take risks, it’s not worth it”; Hikaru moved to a different city without preparation, was self-employed, and enjoyed downhill mountain biking on their day off. In the low-risk condition, Hikaru’s life motto was “There’s no need to worry if you are prepared”; Hikaru had asked friends in their new city to assist in finding a new home and job before moving, had a secure contract, and enjoyed using the fitness bike at the gym on their day off. Apart from these differences, the scenarios were identical in all other aspects (e.g., same city, job description, equal levels of responsibility, and duration).

#### Dominance and prestige

We measured participants’ perceptions of the dominance and prestige of either risk-takers or risk-avoiders. We utilized a validity-assured scale developed by Cheng et al. (2010). The dominance scale comprised eight items (e.g., “Hikaru enjoys having control over others,” “Hikaru often tries to get Hikaru’s way regardless of what others may want to,” “Hikaru is willing to use aggressive tactics to get Hikaru’s way,” “Hikaru does NOT have a forceful or dominant personality [reverse-scored],” “Some people are afraid of Hikaru”). Participants rated these items on a 7-point scale (1 = not at all to 7 = very much so). The Cronbach’s *α* coefficient for this scale was 0.93, indicating an excellent internal consistency.

The prestige scale consisted of nine items (e.g., “Members of Hikaru’s group respect and admire Hikaru,” “Members of Hikaru’s group do NOT want to be like Hikaru” [reverse-scored], “Hikaru’s unique talents and abilities are recognized by others,”). The Cronbach’s α coefficient for this scale was 0.81, indicating a good internal consistency.

#### Leadership endorsement

We measured participants’ contextualized leadership endorsements as utilized by van Kleef et al. (2021; Exp. 3). Participants were asked to indicate their endorsements for the target person across five different leadership roles: leader of a political party, captain of a sports team, representative of real estate associations, CEO of a large oil company, and representative of animal welfare organizations. For each role, participants were queried on the extent to which they would endorse the target person as a leader in both cooperative and competitive intergroup contexts. Items assessing leadership endorsement in cooperative intergroup contexts described situations where cooperative relations with other parties were necessary for resource distribution (e.g., “To what extent would you endorse Hikaru as CEO of a large oil company when cooperation with governments is required to find alternatives for fossil fuels?”). Conversely, items concerning leadership endorsement in competitive intergroup contexts depicted scenarios where the leader would need to advocate for the group’s interests in competition over scarce resources (e.g., “To what extent would you endorse Hikaru as CEO of a large oil company when new oil fields have been discovered and it is crucial to exploit them before other companies do?”). Participants provided responses to the leadership endorsement items using a 7-point scale (1 = not at all, 7 = very much so).

#### Manipulation checks

To assess the efficacy of the risk manipulation, we employed a four-item scale used by van Kleef et al. (2021; Exp.3) (“Hikaru is a risk-taker,” “Hikaru is someone who always plays it safe” [reverse-scored], “Hikaru likes to take gambles,” and “Hikaru tends to choose certainty over uncertainty” [reverse-scored]), rated on a 7-point scale ranging from 1 (not at all) to 7 (very much so). The Cronbach’s *α* coefficient for this scale was 0.97, indicating an excellent internal consistency.

Additionally, we used a context manipulation check developed by van Kleef et al. (2021; Exp. 3) and evaluated the context manipulation by soliciting participants’ opinions on the extent to which each combination of leadership roles and contexts used to gauge contextualized leadership endorsements reflected a cooperative versus competitive situation, using a 7-point scale ranging from 1 (strongly competitive) to 7 (strongly cooperative). The Cronbach’s α coefficient for this measure was 0.97, indicating an excellent internal consistency.

### Procedure

Initially, participants were instructed to read a profile detailing information about a target person and to form an impression of said individual. Subsequently, participants completed three blocks of questions: dominance-related, prestige-related, and leadership endorsement-related questions. The presentation order of these blocks was counterbalanced across participants. Then, participants underwent the risk and context manipulations.

## Results

### Manipulation checks

Participants perceived the target person in the high-risk condition (*M* = 5.67, *SE* = 0.08) as taking significantly more risks than the target person in the low-risk condition (*M* = 1.73, *SE* = 0.07; *t* (297) = 35.67, *p* < 0.001, *d* = 4.13), indicating that the risk manipulation was successful.

To confirm the effectiveness of the context manipulation, we used a linear mixed-effects model, including fixed effects for context, gender, and age, random intercepts for participants and leadership roles, and random slopes for context (see Supplementary file for model selection). No significant main effects were observed for gender (*F* (1, 294) = 2.14, *p* = 0.14) or age (*F* (1, 294) = 0.03, *p* = 0.86), indicating that gender and age differences did not influence the results. However, a significant main effect of context was found (*F* (1, 296) = 766.04, *p* < 0.001). As expected, intergroup cooperative situations were perceived as significantly more cooperative (*M* = 5.36, *SE* = 0.07) than competitive situations (*M* = 2.42, *SE* = 0.08; *t* (296) = −27.68, *p* < 0.001), supporting the validity of the context manipulation.

### Dominance and prestige

**Figure 1:**
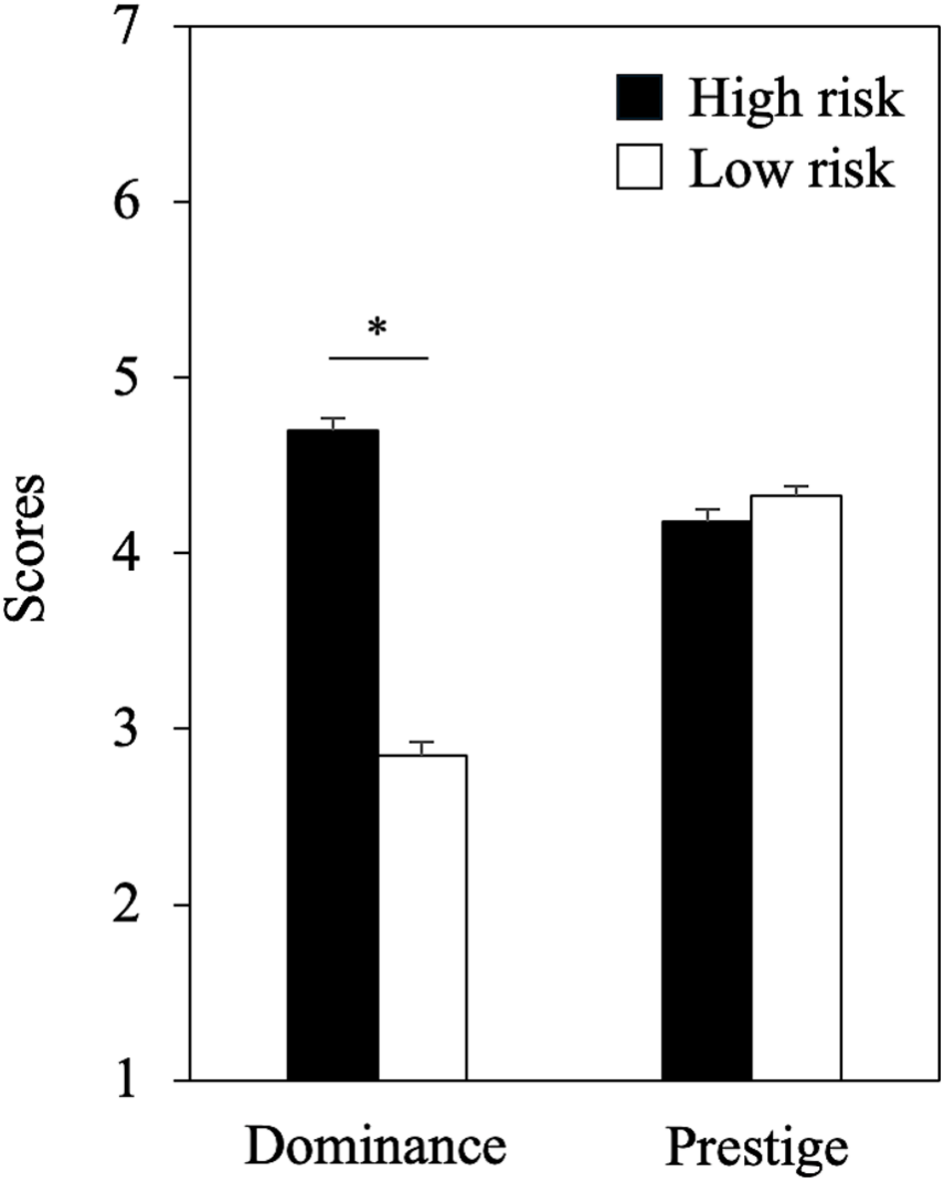
Dominance and prestige scores of the high and low risk conditions. Error bars indicate standard errors. The asterisk indicates *p*-value <0.001.

To investigate whether participants’ evaluations of dominance varied by risk condition, we conducted an Analysis of Covariance (ANCOVA) with the dominance score as the dependent variable. The analysis included two between-subjects factors: condition (high-risk vs. low-risk) and gender (men vs. women), with age included as a covariate. The results revealed no significant effects of gender (*F* (1, 293) = 1.76, *p* = 0.19, *η^2^* = 0.003) or age (*F* (1, 293) = 0.036, *p* = 0.85, *η^2^* = 0.001). However, a significant main effect of condition was observed (*F* (1, 293) = 286.11, *p* < 0.001, *η^2^* = 0.49), indicating that risk-takers were perceived as more dominant than risk-avoiders (*M_risk-taker_* = 4.70, *M_risk-avoider_* = 2.84; *p* < 0.001; Figure 1). A post-hoc power analysis indicated that the ANCOVA achieved a statistical power of 1 − *β* = 1.00 with the given sample size and *α* = 0.05.

To investigate whether participants’ evaluations of prestige varied by risk condition, we conducted an analysis similar to that for dominance. The analysis included prestige score as the dependent variable, with condition and gender as between-subjects factors and age as a covariate. The results showed no significant main effects of gender (*F* (1, 293) = 0.24, *p* = 0.62, *η^2^* = 0.001), age (*F* (1, 293) = 0.008, *p* = 0.93, *η^2^* = 0.001), or condition (*F* (1, 293) = 3.02, *p* = 0.084, *η^2^* = 0.01). Thus, participants did not evaluate risk-takers (*M* = 4.18) and risk-avoiders (*M* = 4.33) differently in terms of prestige (Figure 1). A post-hoc power analysis (referred to as the “Sensitivity” analysis in G*Power) revealed that the ANCOVA, based on the design of the present study, was capable of reliably detecting a small to medium effect size (*f* = 0.16) with the given sample size, *α* = 0.05, and power = 0.8.

### Leadership endorsement

We analyzed the leadership endorsement using a linear mixed-effects model that included fixed effects for condition, context, gender, age, and all two-way interactions, random intercepts for participants and leadership roles, and random slopes for context (see Supplementary file for model selection). There were no significant main effects for condition, context, or gender (*ps* > 0.05), nor were there any significant interactions between condition and gender, condition and age, context and gender, context and age, or gender and age (*ps* > 0.05), indicating that gender and age did not influence the results.

**Figure 2:**
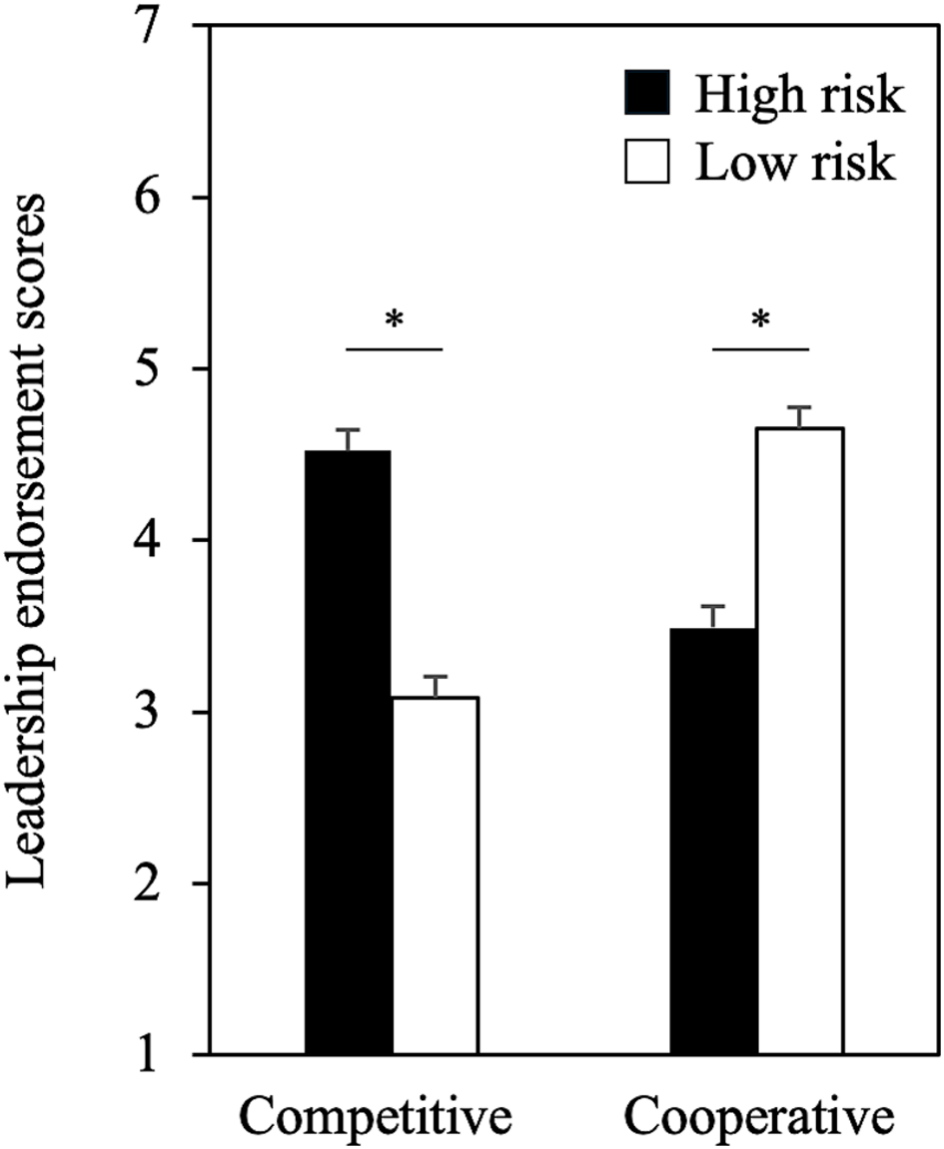
Context-based leadership endorsement of the high and low risk conditions. Error bars indicate standard errors. The asterisk indicates *p*-value <0.001.

A significant interaction was observed between condition and context (*F* (1, 293.01) = 253.69, *p* < 0.001). The interaction pattern revealed that the impact of risk-taking on leadership endorsement varied depending on context. In intergroup competitive contexts, risk-takers were more likely to be endorsed as leaders than risk-avoiders (*M_risk-takers_* = 4.52, *M_risk-avoiders_* = 3.09; *t* (291) = 10.55, *p* < 0.001; Figure 2). In contrast, in intergroup cooperative contexts, participants were more likely to endorse risk-avoiders as leaders than risk-takers (*M_risk-takers_* = 3.49, *M_risk-avoiders_* = 4.65; *t* (291) = −9.11, *p* < 0.001; Figure 2).

### Mediation analysis

**Figure 3:**
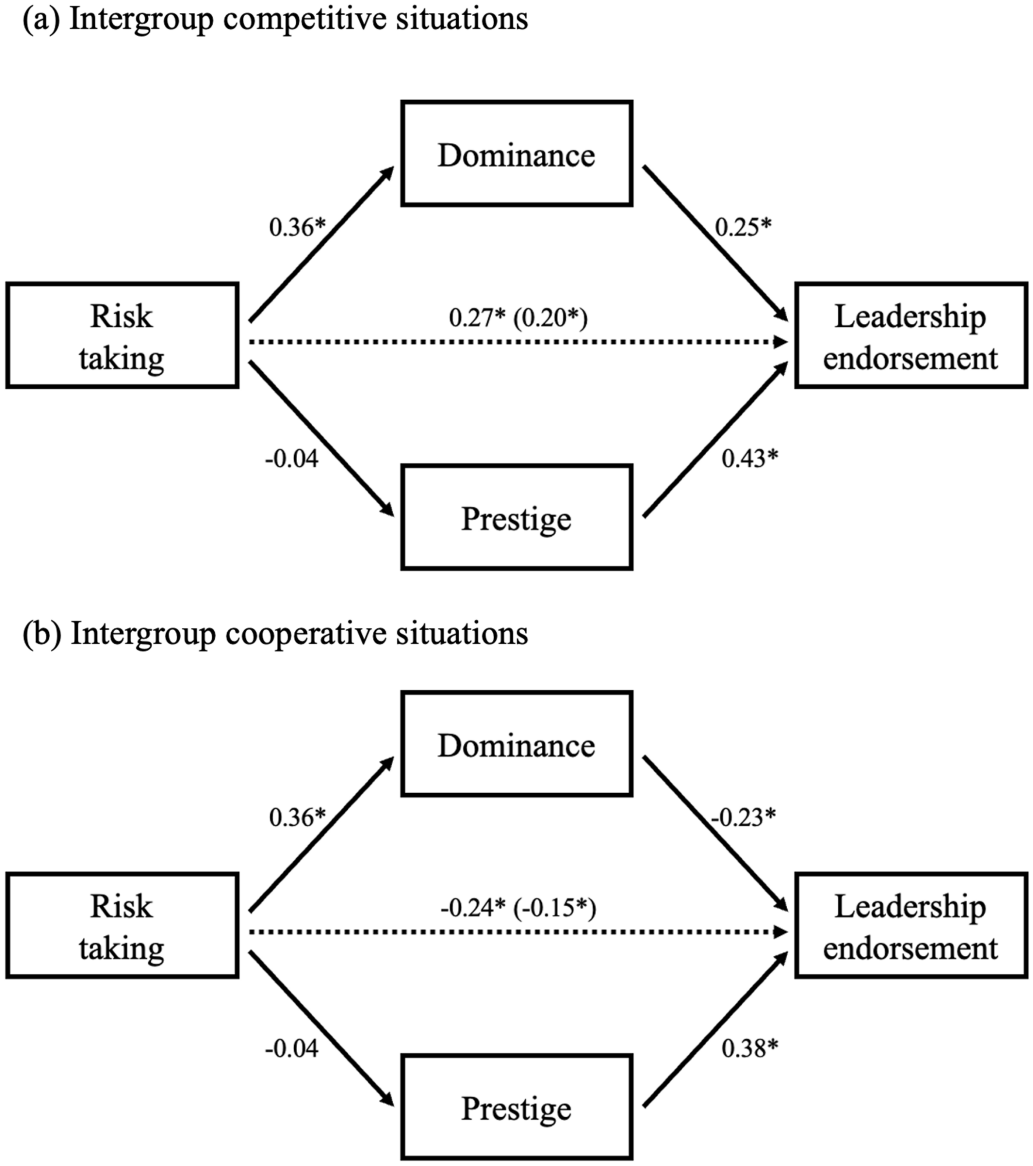
Mediation analysis of the effect of risk-taking on leadership endorsement via dominance and prestige in intergroup competitive **(a)** and cooperative situations **(b)**. Coefficients are standardized estimates. The coefficients for the path from risk-taking to leadership endorsement represent the total effect, with the effect controlling for mediators shown within parentheses. Asterisks indicate significant paths (*p < 0.001).

A mediation analysis was conducted to investigate whether risk-taking influences leadership endorsement in two distinct contexts through the mediating effects of dominance and prestige. The results of a bootstrapping analysis, based on 2,000 bootstrap resamples, revealed a significant indirect effect via dominance in intergroup competitive situations (95% confidence interval [CI]: 0.03, 0.14), whereas no significant indirect effect was observed via prestige (95% CI: −0.05, 0.005). Specifically, risk-taking was associated with increased perceptions of dominance, which in turn enhanced leadership endorsement in intergroup competitive contexts (Figure 3a).

In intergroup cooperative situations, a significant indirect effect via dominance was also identified (95% CI: −0.13, −0.03), while no significant indirect effect was found via prestige (95% CI: −0.04, 0.004). Dominance negatively mediated the relationship between risk-taking and leadership endorsement. Risk-takers were perceived as dominant, which subsequently led to a decrease in leadership endorsement in intergroup cooperative situations (Figure 3b).

## Discussion

The current study demonstrated that participants from Japanese culture perceive risk-takers as more dominant than risk-avoiders; however, no difference was found in their prestige evaluations of these individuals. These are consistent with our Hypothesis 1 and Hypothesis 2. Furthermore, we observed that, compared to risk-avoiders, participants were more likely to endorse risk-takers as leaders in competitive intergroup situations but were less likely to do so in cooperative intergroup contexts, consistent with Hypotheses 3 and 4.

The contrasting evaluation tendencies of risk-takers in terms of prestige between our study and van Kleef et al. (2021) may be explained by cultural variations in regulatory focus. In Japan, there is generally an emphasis on prevention, and individuals may avoid adopting risk-taking strategies to prevent failure or loss (Heine et al., 1999). Consequently, risk-takers may not possess the strategic information that is valued in Japan and may not be regarded as more prestigious than risk-avoiders. From this perspective, one might argue that risk-avoiders could be perceived as having greater prestige than risk-takers, as they are more likely to possess the strategic information valued in Japanese culture. However, in this study, no difference in prestige scores was observed between risk-takers and risk-avoiders. These suggest that in Japan, risk-takers and risk-avoiders are equally valued in terms of prestige, whereas Western cultures may undervalue risk-avoiders compared to risk-takers.

On the other hand, consistent with findings from Western culture (van Kleef et al., 2021), risk-takers were perceived as more dominant than risk-avoiders in Japan. This suggests that in Japan, risk-taking elicits perceptions of dominance through attributes such as increased body size and violence, which may induce feelings of fear in others (Fessler et al., 2014). The cross-cultural similarities in the dominance evaluation of risk-takers raise the possibility that associating risk-taking with dominance has deep roots as an adaptive cognitive bias (van Kleef et al., 2021). Indeed, a study in rodents has shown that risk-taking behavior predicts the emergence of dominant status (Davis et al., 2009). Future research may explore the ontogeny of this cognitive bias to test whether it arises from culture or is a product of natural reasoning.

We conducted a mediation analysis to investigate the mechanisms through which risk-taking influences leadership endorsement across two distinct contexts. Our findings diverged from those of previous research in two key aspects. First, prior research (van Kleef et al., 2021; Exp. 3) found that risk-taking was positively associated with perceived prestige, whereas our study did not reveal this association. This discrepancy may reflect cultural differences in the traits associated with high status between Western and Eastern cultures (Kim and Pettit, 2019; Torelli et al., 2014). In Western cultures, where individual goal attainment and self-actualization take precedence over collective interests (Hofstede, 2001; Markus and Kitayama, 1991; Triandis, 1995), individuals who exhibit traits of extraversion and dominance (e.g., assertiveness, talkativeness, and a tendency to take control) are more likely to attain greater esteem and status (Anderson et al., 2001; Cheng et al., 2013). Risk-takers may be perceived as more assertive than those who avoid risks and, consequently, may be regarded as more prestigious in Western cultures. In contrast, in Eastern cultures such as Japan, where group harmony is emphasized (Hofstede, 2001; Markus and Kitayama, 1991; Triandis, 1995), risk-taking may be perceived as a potential threat to group cohesion. As a result, individuals who take risks may not necessarily be highly regarded.

Second, previous research (van Kleef et al., 2021; Exp. 3) found that perceived dominance did not influence the leadership endorsement of risk-takers in intergroup cooperative situations. In contrast, our study found that perceived dominance negatively influenced the leadership endorsement of risk-takers in such contexts. Participants in Japan, therefore, were hesitant to endorse a risk-taker as a leader in such contexts. In intergroup cooperative settings, endorsing dominant individuals as leaders may be counterproductive, as they tend to prioritize their own preferences over the group’s goals (Maner and Mead, 2010). Previous research has demonstrated that in non-competitive contexts, cooperative individuals are preferred as leaders over dominant ones (Little, 2014). Our findings suggest that, compared to Western cultures, Eastern cultures—such as Japan—are more reluctant to endorse individuals with dominant traits as leaders, particularly when these individuals are likely to disrupt group harmony (Hofstede, 2001; Markus and Kitayama, 1991; Triandis, 1995).

On the other hand, the perceived dominance of the risk-taker increased leadership endorsement of the risk-taker in an intergroup competitive situation. This is consistent with not only the notion that, in intergroup competitive situations, electing a leader capable of effectively resolving conflicts is advantageous for the group (Spisak et al., 2012b), but also that dominant individuals possess the capacity to overcome rivals and foster cooperation by penalizing free riders (Hasty and Maner, 2025). The findings suggest that in Japan, as in Western cultures, risk-takers are favored as leaders in intergroup competitive situations because they are perceived as having the necessary skills to navigate the competition.

### Limitation

Our study has several limitations that should be addressed in future studies. First, it should be noted that our results may reflect attitudes toward personal values rather than risk-taking per se. In both the previous study (van Kleef et al., 2021) and the current study, risk-takers in the presented scenarios were portrayed as highly focused on personal accomplishments and hobbies, with minimal emphasis on collectivist achievements or family and community relationships. These characteristics align with the values of predominantly individualistic cultures (Markus and Kitayama, 1991). In other words, findings from Western culture (van Kleef et al., 2021) may reflect positive evaluations of individuals with individualistic attitudes (e.g., risk-takers in the scenarios), regardless of whether they engaged in risk-taking behavior. In contrast, collectivist societies, such as those in Eastern cultures, which prioritize group harmony (Markus and Kitayama, 1991), may negatively evaluate individuals who exhibit individualistic attitudes. In other words, collectivist societies may place a higher value on individuals who embody collectivist attitudes. In the present study, the absence of a difference in the evaluation of prestige between risk-takers and risk-avoiders may be attributed to the positive evaluation of risk-avoiders in Eastern cultures. Future research should examine social evaluations of risk-takers in contexts unrelated to individualistic attitudes, such as personal interests.

Second, while this study suggested that some social evaluations of risk-takers are influenced by culture, it should be noted that the study was conducted exclusively with Japanese participants and did not directly compare data across cultures. Thus, our study could not clarify the extent of cultural differences between the East and the West. For example, in our study, Japanese participants did not perceive risk-takers as more prestigious than risk-averse individuals. However, we were unable to determine the degree to which this evaluation of prestige toward risk-takers differs when compared to Western cultures. Future studies should include samples from both Western and Japanese populations to enable direct cross-cultural comparisons.

Third, our study did not identify the psychological mechanisms underlying differences in prestige ratings of risk-takers across cultures (van Kleef et al., 2021). Addressing this limitation will require future research to incorporate factors that explain these cultural differences. One potential framework is regulatory focus theory (Higgins, 1997, 2000). Previous researches have shown that Westerners are more aligned with a promotion focus, while Easterners are more aligned with a prevention focus (Heine et al., 2001; Lee et al., 2000; Lockwood et al., 2005). Westerners may evaluate risk-takers more positively and as more prestigious because their regulatory focus aligns with the behavior of risk-takers. Conversely, Easterners may evaluate risk-takers more negatively and as less prestigious because risk-taking behavior does not align with their regulatory focus. Testing these potential mechanisms would provide a more comprehensive understanding of the cultural dynamics at play.

## Data Availability

The raw data supporting the conclusions of this article will be made available by the authors, without undue reservation.

## Footnotes

^1^Based on reviewer suggestions, we determined that ANCOVA was more appropriate than an independent-samples t-test, as it allows for testing the potential effects of age and gender by treating them as covariates. Post-hoc power analyses, which indicate the power or sensitivity of the tests, have been reported for both prestige and dominance evaluations.
