# Supplementary material for: Risk-taking influences perceived dominance, prestige, and leadership endorsement in Japanese adults
Source: Front Psychol. 2025 Apr 23;16:1529892. doi: 10.3389/fpsyg.2025.1529892 (PMC12055805; doi:10.3389/fpsyg.2025.1529892)
Supplement: Supplementary file 2 [file Presentation_1.PDF]

Supplementary file for

Risk-taking influences perceived dominance, prestige, and leadership in Japanese Adults

This supplementary information includes:

- Profiles of target individuals in Japanese and English versions: High-risk vs. low-risk condition
- Model selection in the analysis of context manipulation check and leadership Endorsement

Profiles of target individuals in Japanese and English versions: High-risk vs. low-risk condition

■ Japanese Version:

(a) High-risk condition

名　　前：ヒカル

年　　齢：29 歳

座右の銘：「リスクを冒さなければ価値はなし」

自己紹介：ヒカルはＡ県の大学卒業後、すぐにＢ県でコンサルタントとして働き始めました。そこで４年間働きましたが、何か違うことをしたいと考えるようになりました。そこでＡ県に戻ることを思い付き、すぐに仕事を辞め、アパートを解約し、なんの準備もなくＡ県に引っ越しました。しばらくして、ヒカルは、Ａ県の会社がマーケティングの従業員を募集していることを知りました。そして、その会社でヒカルはフリーランスとして契約してもらうことにしました。現在は、会社から独立し、マーケターとして２年半働いており、２人の従業員を雇用しています。ヒカルは起業家として、起業をするならそれに伴うリスクを負うことは必要だと考えています。それは、ヒカルが、「結局未来のことは誰もわからないし、経営者として安全策を取るよりも大きなリスクを負うことで、より大きな利益を生むことができる」と考えているからです。また、ヒカルは仕事の息抜きにスポーツをすることが好きで、怪我をするかもしれないがスリルのあるスポーツをしたいと思っています。なので、休日には車にバイクを積んで山に出かけ、山道をバイクで下るスポーツを楽しんでいます。

(b) Low-risk condition

名　　前：ヒカル

年　　齢：29 歳

座右の銘：「備えあれば憂いなし」

自己紹介：ヒカルは A 県の大学卒業後、すぐに B 県でコンサルタントとして働き始めました。そこで 4 年間働きましたが、何か違うことをしたいと考えるようになりました。そこで A 県に戻ることを思い付きました。ヒカルは、引っ越

しをする前に住む家と仕事が決まっていれば安心だと思ったので、A 県に住む友人や知人に連絡をとり、予め居住先や仕事について調べてもらいました。引っ越し後すぐに、A 県の会社のマーケティング部署で働くことになりました。現在は、その会社のマーケターとして 2 年半勤務しています。またマネージャーになり、2 人の部下と共に働いています。ヒカルはマネージャーとして、常に注意深く、できる限りリスクを避けるように心がけています。それは、ヒカルが、「結局未来のことは誰もわからないし、マネージャーとしてリスクを負うよりも安全策を取ることで、大きな損失を避けることができる」と考えているからです。また、ヒカルは仕事の息抜きにスポーツをすることが好きですが、できる限り怪我をせず安全なスポーツをしたいと思っています。なので、休日にはジムに行ってフィットネスバイクで運動をすることを楽しんでいます。

■ **English Translation:**

(a) High-risk condition

Name: Hikaru

Age: 29 years old

Motto: "If you don't take risks, it's not worth it"

Self-introduction: After graduating from a university in Prefecture A, Hikaru immediately started working as a consultant in Prefecture B. Hikaru worked there for four years but began to feel like Hikaru wanted to do something different. So, Hikaru came up with the idea of returning to Prefecture A, quitting his job right away, canceling his apartment lease, and moving to Prefecture A without any preparation. After a while, Hikaru learned that a company in Prefecture A was recruiting marketing employees. Hikaru decided to contract with the company as a freelancer. Currently, Hikaru has been working as a marketer for two and a half years, having become independent from the company and employing two staff members. Hikaru believes that as an entrepreneur, it is necessary to take risks associated with starting a business. This is because Hikaru believes that ultimately, no one knows the future, and by taking bigger risks as a business owner rather than playing it safe, one can generate greater profits. Additionally, Hikaru enjoys sports as a way to unwind from work and wants to engage in thrilling sports that might involve getting injured. Therefore, on weekends, Hikaru loads his bike onto his car and heads to the

mountains to enjoy the sport of biking down mountain trails.

(b) Low-risk condition

Name: Hikaru

Age: 29 years old

Motto: “There’s no need to worry if you’re prepared”

Self-introduction: After graduating from a university in Prefecture A, Hikaru immediately started working as a consultant in Prefecture B. Hikaru worked there for four years but began to feel like Hikaru wanted to do something different. So, Hikaru came up with the idea of returning to Prefecture A. Hikaru thought it would be reassuring if Hikaru had a place to live and a job lined up before moving, so Hikaru contacted friends and acquaintances in Prefecture A to research housing and employment in advance. Soon after moving, Hikaru found a job in the marketing department of a company in Prefecture A. Hikaru has been working there as a marketer for 2 and a half years. Hikaru has also become a manager and works alongside two subordinates. As a manager, Hikaru always strives to be careful and avoid risks as much as possible. This is because Hikaru believes that, ultimately, no one knows the future, and as a manager, avoiding risks rather than taking them can prevent major losses. Additionally, while Hikaru enjoys sports as a way to unwind from work, Hikaru prefers to engage in safe sports to minimize the risk of injury. Therefore, on weekends, Hikaru enjoys going to the gym and exercising on a fitness bike.

Model selection in the analysis of context manipulation check and leadership Endorsement

The study by van Kleef et al. (2021) predominantly comprised women (115 women and 34 men), with all participants being psychology students. In contrast, the present study included a more balanced gender ratio, with participants being nearly twice as old as those in the van Kleef et al. (2021) sample. Additionally, the majority of participants in the current study were not students.

To account for these differences, we included gender and age as fixed effects in

the linear mixed-effects model used in van Kleef et al. (2021; Exp. 3) for the analysis of the context manipulation check and leadership endorsement. However, our model differed from the previous study, as gender and age were not included as factors in their original analysis. Consequently, we first needed to identify the best-fitting model by determining which interactions should be included in the fixed effects.

■ Model selection for context manipulation check

To identify the most appropriate model for the contextual manipulation data, we compared three linear mixed-effects models using the Akaike Information Criterion (AIC), Bayesian Information Criterion (BIC), and Likelihood Ratio Test (LRT). The simplest model (Model 1) included fixed effects for context, gender, and age, along with random intercepts for participants and leadership roles, as well as random slopes for context. Model 2 extended Model 1 by incorporating all two-way interactions, while Model 3 further included a three-way interaction.

The AIC and BIC values for each model were as follows: Model 1 (AIC = 9633.9, BIC = 9687.8), Model 2 (AIC = 9638.4, BIC = 9710.4), and Model 3 (AIC = 9640.4, BIC = 9718.3). LRTs were conducted to assess the relative fit of the models. The comparison between Model 1 and Model 2 revealed no significant difference in fit (χ^2 (3) = 1.46, p = .69). Similarly, the comparison between Model 2 and Model 3 indicated no significant improvement (χ^2 (1) = 0.002, p = .97). These findings suggest that Model 2 and Model 3 do not provide a statistically superior fit compared to Model 1.

Considering the AIC and BIC values, as well as the lack of significant improvement in model fit for the more complex models, Model 1 is identified as the most appropriate choice for subsequent analyses due to its parsimony and comparable fit.

■ Model selection for leadership endorsement

To identify the most appropriate model for the leadership endorsement data, we compared four linear mixed-effects models using the Akaike Information Criterion (AIC), Bayesian Information Criterion (BIC), and Likelihood Ratio Test (LRT). The simplest model (Model 1) included fixed effects for condition, context, gender, and age, along with random intercepts for participants and leadership roles, as well as random slopes for

contexts. Model 2 extended Model 1 by incorporating all two-way interactions. Model 3 further included all three-way interactions, while Model 4 incorporated a four-way interaction.

The AIC and BIC values for each model were as follows: Model 1 (AIC = 9938.0, BIC = 9998.0), Model 2 (AIC = 9761.6, BIC = 9857.6), Model 3 (AIC = 9765.7, BIC = 9885.6), and Model 4 (AIC = 9767.6, BIC = 9893.5). LRTs were conducted to assess the relative fit of the models. The comparison between Model 1 and Model 2 revealed a significant improvement in model fit (χ^2 (6) = 188.40, $p < .001$, $\Delta AIC = -176.4$). However, the inclusion of three-way (Model 3) or four-way (Model 4) interactions did not yield further improvements in model fit (Model 2 vs. Model 3: χ^2 (4) = 3.91, $p = .42$; Model 3 vs. Model 4: χ^2 (1) = 0.12, $p = .73$). Based on these results, Model 2 was identified as the best-fitting model.
